# Unveiling the role of TRPA1 in cardiovascular health and disease: a mini review

**DOI:** 10.3389/fcvm.2024.1416698

**Published:** 2024-09-11

**Authors:** Islam Gellani, Chunqi Qian, Shuangtao Ma

**Affiliations:** ^1^ Department of Medicine, College of Human Medicine, Michigan State University, East Lansing, MI, United States; ^2^ Department of Radiology, College of Human Medicine, Michigan State University, East Lansing, MI, United States

**Keywords:** TRPA1, ion channel, cardiovascular disease, myocardial infarction, myocardial fibrosis

## Abstract

The transient receptor potential ankyrin 1 (TRPA1) ion channel has emerged as significant regulators of cardiovascular physiology and pathology. TRPA1 is a non-selective cation channel permeable to calcium ions. A unique feature of the channel is its function as a sensor of various temperature, chemical and mechanical stimuli, while it can also be activated by endogenous inflammatory mediators and reactive oxygen species. Over the last two decades, much progress has been made in illuminating the role of TRPA1 in the regulation of cardiovascular physiology and pathophysiology in addition to its important function in pain sensation. This review provides a comprehensive analysis of recent studies investigating the involvement of TRPA1 channels in various cardiovascular diseases, including myocardial infarction, ischemia-reperfusion injury, myocardial fibrosis, and response to environmental toxins. We discuss the diverse roles of TRPA1 channels in cardiac pathology and highlight their potential as therapeutic targets for cardiovascular disorders. Moreover, we explore the challenges and opportunities linked with targeting TRPA1 channels for treating cardiovascular diseases, alongside future research directions.

## 1 Introduction

Cardiovascular diseases remain a leading cause of mortality worldwide, necessitating a deeper understanding of their underlying mechanisms and the development of novel therapeutic strategies. Recent studies have implicated transient receptor potential ankyrin 1 (TRPA1) channels, known for their role as sensory receptors (1), in the pathogenesis of cardiovascular diseases (2–4). Found predominantly in nociceptive sensory neurons, TRPA1 channels belong to the transient receptor potential (TRP) ion channel superfamily, known for their role in sensory transduction, notably in pain sensation (5). TRPA1 and TRP melastatin 8 are considered as sensors of cold and cool temperatures, respectively (6–8), and they are involved in the vascular sensation of cold in the cutaneous microvasculature (9, 10). These channels serve as sensitive detectors of various stimuli, orchestrated through specialized receptors decorating the neuronal cell membrane (11, 12). TRPA1 is involved in the pathophysiology of a variety of diseases (13, 14), including renal diseases (15, 16), and clinical trials are currently testing the therapeutic effects of its agonists and antagonists in various neurological and dermatological disorders (17). TRPA1 channels are expressed in various cardiovascular cells, including vascular endothelial cells, vascular smooth muscle cells, cardiac fibroblasts, as well as cardiomyocytes (18, 19).

Structurally, TRPA1 channels exhibit a sophisticated architecture consisting of six transmembrane domains (TM), bordered by intracellular NH2 and COOH termini, with a pivotal pore-forming loop nestled between TM5 and TM6 (20–22). Of particular note is the expansive NH2 terminus, housing 14 (in mice) or 16 (in humans) ankyrin repeat domains, important for protein-protein interactions and imparting structural elasticity (20). Functionally, TRPA1 channels exhibit remarkable sensitivity to a wide range of triggers, facilitated by the presence of specialized receptors on the neuron's cell membrane (1, 23–25). TRPA1 can be covalently and non-covalently activated by various noxious and reactive chemical compounds (25, 26). For example, plants-derived reactive electrophiles such as allyl isothiocyanate (AITC) and cinnamaldehyde activate TRPA1 by covalently binding to its N-terminal cytoplasmic cysteine residues while nonreactive compounds such as menthol and thymol activate TRPA1 without covalent binding (27). By carefully analyzing mutagenesis and deletion constructs, researchers have pinpointed crucial regions that control various functional aspects of TRPA1 channels. These regions regulate calcium sensitivity, responsiveness to electrophiles, phosphorylation, rectification, conductance, and sensitivity to agonists such as AITC and carvacrol. Abnormalities within the realm of TRPA1 lead to specific functional changes, highlighting its contribution to nociception and disorders related to pain. This underscores the critical role TRPA1 plays in sensory transduction. Activation of TRPA1 channels has been shown to influence vascular tone (28, 29), inflammation (16, 30), oxidative stress (29), and cardiac remodeling (31), thereby impacting cardiovascular health. This review focuses on the role of TRPA1 in the development of cardiovascular diseases rather than the underlying regulatory molecular mechanisms as they have been discussed in previous reviews (2, 3).

## 2 Role of TRPA1 in ischemic heart disease: insights and therapeutic implications

Ischemic heart disease is myocardial injury caused by insufficient coronary blood flow to the heart. Persistent acute myocardial ischemia for a prolonged period can lead to irreversible injury or necrosis of cardiomyocytes, resulting in myocardial infarction (MI). This condition typically results from the obstruction of coronary arteries, often due to rupture of atherosclerotic plaques and subsequent formation of thrombosis. Myocardial ischemia/reperfusion (I/R) injury, resulting from the restoration of blood flow after ischemia, involves complex cellular and molecular processes. Ischemia initiates intracellular and extracellular changes, including calcium overload and ATP depletion, resulting in cellular dysfunction and death. Upon reperfusion, immediate effects like mitochondrial pore permeability and cytokine release exacerbate tissue damage, leading to long-term consequences and progressive cardiomyocyte death (32).

In the context of ischemic heart disease, the role of TRPA1 has garnered considerable interest. In recent studies focusing on the role of TRPA1 channels in MI and myocardial I/R injury, researchers have uncovered valuable insights into the molecular mechanisms underlying this pathological process and its potential therapeutic implications. However, the conclusions from these studies are highly variable.

First of all, a genome-wide association study revealed that TRPA1 variant at rs12541758 is the strongest susceptibility allele for coronary artery disease in humans, which provided the most conspicuous association between TRPA1 and ischemic heart disease (33). TRPA1 channels could be protective against myocardial ischemic injury according to studies. In a study conducted by Lu et al. (34), the presence and role of TRPA1 channels in cardiac myocytes and their involvement in regulating myocardial I/R injury were investigated. Using biochemical analysis techniques and a rat model of cardiac I/R injury, the researchers demonstrated that TRPA1 is indeed present within cardiac myocytes. Activation of TRPA1 reduced myocardial injury in the rat model, while inhibition of TRPA1 blocked the infarct size-sparing effects of morphine, indicating a potential interaction between TRPA1 signaling and analgesic pathways. In isolated cardiac myocytes subjected to hypoxia-reoxygenation, TRPA1 activation reduced cell death during reoxygenation, further supporting the role of TRPA1 in modulating cellular responses to I/R injury (35). However, the beneficial effects of TRPA1 agonists were not observed by Hoebart et al. who conducted a study to unravel the involvement of TRPA1 in MI and I/R injury (36). Through a series of experiments utilizing both pharmacological and genetic approaches, the researchers illuminated potential implications of TRPA1 in myocardial injury and sensory neuron-cardiomyocyte interactions. In pharmacological modulation studies conducted on Sprague-Dawley rats, the administration of TRPA1 agonists and antagonists did not significantly impact infarct size post-I/R injury. Similarly, genetic deletion of TRPA1 in C57BL/6J mice did not yield substantial differences in infarct size compared to wild-type mice. These findings, while suggestive of a potential role for TRPA1, did not establish a direct link between TRPA1 modulation and MI *in vivo*. *In vitro* experiments involving sensory neurons derived from dorsal root ganglia and cardiomyocytes provided intriguing insights into the interplay between TRPA1 and cardiomyocyte survival under I/R conditions. While the presence of sensory neurons enhanced cardiomyocyte survival, the specific involvement of TRPA1 in this process remained inconclusive. Additionally, investigations into the role of another sensory neuron receptor, TRP vanilloid 1, did not significantly alter cardiomyocyte protection under IR conditions, further emphasizing the complexity of sensory neuron-cardiomyocyte interactions.

In contrast, some studies demonstrated that TRPA1 channels could be detrimental to MI and myocardial I/R injury. Conklin and colleagues investigated the contribution of TRPA1 channels to myocardial I/R injury using mouse models, including both wild-type and Trpa1 gene knockout (*Trpa1*^−/−^) mice (37). Their findings revealed that *Trpa1*^−/−^ mice exhibited significantly smaller infarct sizes following I/R compared to WT mice. Additionally, immunofluorescence staining revealed the presence of TRPA1 channels in both murine and human hearts, with notable enrichment observed in the intercalated disks of cardiomyocytes. Further experiments using isolated cardiomyocytes showed that TRPA1 deficiency conferred resistance to acrolein-induced toxicity, a known activator of TRPA1 channels. Blocking TRPA1 with a specific antagonist protected cardiomyocytes from acrolein-induced calcium overload and hypercontraction, suggesting a role for TRPA1-mediated calcium dysregulation in myocardial injury during I/R. These findings highlight TRPA1 as a potential therapeutic target for mitigating myocardial I/R injury, potentially through modulation of calcium homeostasis and cellular toxicity pathways.

Studies demonstrated that inhibition of TRPA1 might promote myocardial repair after MI. In a study by Li et al., TRPA1's potential as a therapeutic target for improving outcomes post-MI was explored using murine models (38). Male C57BL/6 mice subjected to MI injury exhibited increased TRPA1 expression, implicating TRPA1 in MI-induced cardiac injury. Treatment with the TRPA1 inhibitor HC-030031 (HC) post-MI resulted in improved cardiac function, reduced myocardial apoptosis, and ameliorated pathological changes, including reduced infarct size and cardiac fibrosis. Mechanistic insights revealed that TRPA1 inhibition promoted angiogenesis and activated the phosphoinositide-3 kinase/protein kinase B signaling pathway, offering potential avenues for therapeutic intervention in MI.

Overall, while the exact role of TRPA1 in ischemic heart disease remains complex and multifaceted, these studies collectively contribute to our understanding of TRPA1's involvement in MI and myocardial I/R injury and offer potential therapeutic targets for mitigating myocardial damage and improving outcomes of MI. The controversial results among studies might be due to the complicated role of TRPA1 in regulating cellular injury and defense during myocardial ischemia. Many factors could be responsible for the contradictory results. The role of TRPA1 may vary from different animal models and may be distinct at different stages of myocardial ischemia and MI. In addition, TRPA1 channels are expressed in various cell types in the heart, including cardiomyocytes, sensory nerve endings, fibroblasts, and macrophages. Future studies using conditional Trpa1 gene knockout mouse models may be helpful to further characterize its role in MI and I/R injury. Possible nonspecific actions of TRPA1 agonists should also be considered and ruled out using specific antagonists or gene knockout mouse models.

## 3 TRPA1 channels in myocardial fibrosis

Myocardial fibrosis, characterized by the excessive accumulation of collagen and other extracellular matrix proteins in the heart muscle, represents a hallmark feature of congestive heart failure pathogenesis. There are two types of myocardial fibrosis: reactive fibrosis and reparative. Reactive fibrosis refers to interstitial fibrosis mostly due to hypertension or aging, while reparative fibrosis, also known as replacement fibrosis, is responsible for cardiac wound healing and repair after MI. Reactive fibrosis is likely associated with decreased degradation of collagens, whereas reparative fibrosis is mainly due to activation of fibroblasts and increased synthesis of collagens (39, 40). Recent research has shed light on the role of TRPA1 channels in modulating myocardial fibrosis and cardiac dysfunction in response to different pathological stimuli. Studies utilizing animal models and experimental approaches have provided valuable insights into the potential therapeutic implications of targeting TRPA1 in the context of myocardial fibrosis.

Our recent study, utilizing TRPA1 gene knockout mice, has linked TRPA1 to age-related cardiac remodeling and dysfunction have also been linked to TRPA1 (31). We used 12-week-old (young) and 52-week-old (older) *Trpa1*^−/−^ mice along with WT littermates as their animal models. We evaluated cardiac function and pathology using echocardiography and histological analyses and measured the expression levels of 84 fibrosis-related genes in heart tissue. Our findings reveal that older *Trpa1*^−/−^ mice exhibited significantly increased left ventricular internal diameter and volume, impaired systolic and diastolic functions, and enhanced cardiac fibrosis compared to older WT mice, despite similar levels of cardiac hypertrophy between the two strains. Analysis of fibrosis-related genes showed significant upregulation of pro-fibrotic genes and downregulation of anti-fibrotic genes in older *Trpa1*^−/−^ mice compared to older WT mice. These results suggest that TRPA1 deficiency exacerbates age-related myocardial fibrosis, ventricular dilation, and cardiac dysfunction, indicating a potentially anti-fibrotic role of TRPA1 in the context of cardiac aging. The exacerbation of myocardial fibrosis and ventricular dysfunction in older *Trpa1*^−/−^ mice suggests a protective role of TRPA1 against age-related cardiac pathology (31). In doxorubicin-induced dilated cardiomyopathy rat model, activation of TRPA1 using its agonist cinnamaldehyde alleviated cardiac dysfunction and fibrosis, suggesting a protective role of TRPA1 in myocardial fibrosis and dilated cardiomyopathy (41). Similarly, another study demonstrated that activating TRPA1 attenuates right ventricular fibrosis in a model of monocrotaline-induced pulmonary arterial hypertension through secretion of calcitonin gene-related peptide (42). These findings imply that interventions aimed at enhancing TRPA1 activity or expression may have therapeutic potential for preventing or slowing down myocardial fibrosis and associated cardiovascular complications.

On the other hand, studies highlighted pro-fibrotic effects of TRPA1 in the process of replacement cardiac fibrosis. For instance, Li et al. investigated the role of TRPA1 in driving cardiac myofibroblast transdifferentiation post-MI injury (43). Their findings suggest that TRPA1 activation promotes cardiac myofibroblast transdifferentiation via the calcineurin/nuclear factor of activated T cell signaling pathway. These results imply that targeting TRPA1-mediated myofibroblast activation may represent a potential therapeutic strategy for reducing post-MI fibrotic remodeling and improving cardiac function in MI patients. In a rat model of diabetic cardiomyopathy, Wang et al. demonstrated that TRPA1 deficiency exerts protective effects against cardiac dysfunction and fibrosis (44). Their findings suggest that TRPA1 inhibition may represent a promising therapeutic strategy for mitigating myocardial fibrosis in diabetic patients, potentially reducing the risk of congestive heart failure, and improving overall cardiac function. The identification of the G protein-coupled receptor kinase 5 and nuclear factor of activated T cell signaling pathway as a downstream mediator of TRPA1-related fibrotic changes highlights specific molecular targets for future drug development efforts aimed at modulating TRPA1 activity. In the context of pressure overload-induced cardiac hypertrophy and fibrosis, Wang et al. demonstrated that TRPA1 inhibition attenuates cardiac remodeling and fibrosis post-transverse aortic constriction surgery (45). This suggests that pharmacological targeting of TRPA1 may represent a novel approach for managing pressure overload-induced cardiac diseases, such as hypertrophic cardiomyopathy and congestive heart failure. The identification of calcium-dependent signaling pathways and M2 macrophage transition as mechanisms underlying the protective effects of TRPA1 inhibition provides valuable insights into the complex molecular pathways involved in myocardial fibrosis regulation.

In conclusion, the collective findings from these studies highlight the significance of TRPA1 modulation in myocardial fibrosis and associated cardiac dysfunction. The TRPA1 variant rs574328516 is related to cardiomyopathy in humans according to FinnGen Data Freeze 10 (https://r10.finngen.fi). The role of TRPA1 in cardiac fibrosis varies in different contexts. Overall, TRPA1 channels likely inhibit reactive fibrosis but stimulate reparative fibrosis. Targeting TRPA1 signaling pathways holds promise for developing novel therapeutic interventions aimed at preventing or mitigating myocardial fibrosis-related cardiovascular complications. These diverse findings of previous studies underscore the complexity of TRPA1 signaling in fibrosis regulation and highlight the need for further research to understand its precise role and therapeutic potential in different pathological conditions. Further research is needed to clarify the exact molecular mechanisms underlying TRPA1-mediated fibrotic remodeling and to translate these findings into clinically relevant therapeutic strategies for improving patient outcomes in cardiovascular diseases.

## 4 TRPA1 agonists: implications for cardiovascular responses

The investigation into the cardiovascular effects of TRPA1 agonists, particularly AITC and cinnamaldehyde, has revealed significant findings with broad implications for cardiovascular physiology and potential therapeutic strategies. Studies have demonstrated that TRPA1 activation by AITC and cinnamaldehyde elicits diverse cardiovascular responses in animal models. In normotensive rats, AITC inhalation evoked atropine-sensitive bradycardia, indicative of parasympathetic reflex activation, whereas spontaneously hypertensive rats exhibited complex brady-tachycardia accompanied by atrial-ventricular block and premature ventricular contractions (46). Furthermore, intravenous administration of AITC induced bradycardia in both rat models, indicating distinct reflex pathways depending on the route of administration (46). Additionally, investigations into cinnamaldehyde's cardiovascular effects revealed its ability to induce relaxation of rat aortic rings and negative inotropic and chronotropic effects on isolated mouse hearts (47). *In vivo* mouse experiments demonstrated that cinnamaldehyde could induce a systemic depressor response through activating TRPA1 (4) and increase cerebrovascular blood flow by mediating a neurogenic vasodilatation (48).

These findings have several implications for cardiovascular health. Firstly, it suggests that modulation of TRPA1 channels may offer novel strategies for managing hypertension, ischemic heart disease, and peripheral vascular disorders. Another way is that the inhibition of L-type calcium currents by TRPA1 agonists suggests a potential role for TRPA1 channels in modulating cardiac contractility and electrical activity, which could lead to the development of selective TRPA1 modulators or channel blockers for managing cardiac arrhythmias and heart failure. The involvement of TRPA1 agonists in cardiovascular reflexes suggests a potential crosstalk between nociceptive and cardiovascular pathways, providing insights into the pathogenesis of conditions characterized by both pain and cardiovascular dysfunction. Further research in this area may uncover additional therapeutic targets and strategies for improving cardiovascular health and treating related disorders. Furthermore, gaining a deeper understanding of the exact mechanisms through which TRPA1 agonists exert their cardiovascular effects holds the potential to pave the way for the development of more targeted and effective therapeutic interventions. Such advancements have the promise of significantly enhancing outcomes for patients grappling with cardiovascular disease

## 5 TRPA1 in cardiovascular health: response to environmental toxins

TRPA1 is a versatile protein known for its involvement in various physiological processes, including sensory perception and vasoreactivity. Particularly relevant to cardiovascular health, TRPA1 has emerged as a critical mediator in response to environmental toxins, such as crotonaldehyde and acrolein, commonly found in cigarette smoke and air pollution. Understanding the intricate interplay between TRPA1 activation and cardiovascular outcomes in the context of toxin exposure is essential for deciphering mechanisms underlying toxin-induced cardiovascular toxicity and identifying potential therapeutic targets.

Studies by Jin et al. have shed light on the cardiovascular toxicity of crotonaldehyde, an abundant component of cigarette smoke, and its interaction with TRPA1 (49, 50). Both acute and chronic exposure to crotonaldehyde led to hypotension and augmented relaxations in vascular tissue, with TRPA1 playing a pivotal role in mediating these effects (49, 50). In *Trpa1*^−/−^ mice, crotonaldehyde exposure had no significant impact on aortic function, highlighting the involvement of TRPA1 in crotonaldehyde-induced cardiovascular effects (49, 50). These findings suggest that TRPA1 activation may represent a key mechanism underlying crotonaldehyde-induced cardiovascular toxicity, emphasizing its potential as a therapeutic target for mitigating the adverse cardiovascular effects of cigarette smoke exposure. Similarly, acrolein, a highly toxic aldehyde present in tobacco smoke and air pollution, has been implicated in cardiovascular toxicity, with TRPA1 playing a crucial role in mediating its effects (51). Studies by Conklin et al. demonstrated that both male and female mice lacking the TRPA1 gene exhibited increased susceptibility to acrolein-induced mortality compared to wild-type mice, suggesting a protective role of TRPA1 (52, 53). Surprisingly, post-acrolein exposure treatment with a TRPA1 antagonist (HC030031) provided significant protection against acrolein-induced mortality (52, 53). Further investigation needs to address the compensatory effects of TRPA1 upon acrolein exposure and to reveal if the protective effects of HC030031 are mediated by TRPA1 inhibition. Additionally, investigations by Kurhanewicz et al. revealed that exposure to acrolein led to cardiac autonomic imbalance and arrhythmia in wild-type mice (54, 55), which was absent in *Trpa1*^−/−^ mice, underscoring the crucial role of TRPA1 in mediating the cardiovascular effects of acrolein exposure. TRPA1 was also reported to mediate diesel exhaust exposure-induced hypersensitivity of cardiac arrhythmias (56). Ethanol and its metabolite acetaldehyde are known as cardiac toxins, and they can also activate the TRPA1 channel (53, 57). However, it is unclear whether TRPA1 plays a role in the effects of ethanol intake on the cardiovascular system.

Overall, these studies collectively highlight the pivotal role of TRPA1 in mediating cardiovascular responses to environmental toxins, such as crotonaldehyde and acrolein. Understanding the mechanisms underlying TRPA1 activation in response to toxin exposure is crucial for identifying novel therapeutic strategies aimed at mitigating toxin-induced cardiovascular toxicity. Future research efforts should focus on elucidating the intricate signaling pathways and downstream effects of TRPA1 activation in cardiovascular tissues, with the ultimate goal of developing targeted interventions to protect against toxin-induced cardiovascular damage and promote cardiovascular health in toxin-exposed populations.

## 6 Potential mechanisms underlying the role of TRPA1

The exact mechanism underlying the regulatory role of TRPA1 in cardiovascular health and disease is not well understood and is quite controversial. TRPA1 potentially has direct and/or indirect effects on myocardium. Studies have reported that the roles of TRPA1 are likely mediated by its effects on oxidative stress and inflammation (41). However, the direct effects of TRPA1 remain questionable as a recent study reported that no functional TRPA1 is expressed in cardiomyocytes, which makes a direct effect in cardiomyocytes unlikely (58). It has been confirmed that TRPA1 is abundantly expressed in sensory nerve endings including vagal afferent nerve fibers and that activation of TRPA1 stimulates vagal nerve activity (59–61). Improved vagal nerve activity can exert anti-inflammatory actions through binding to alpha 7 nicotinic acetylcholine receptor on macrophages and other immune cells (59, 62). So far, the indirect effects of TRPA1 through regulating cardiac vagal tone seem to be reasonable but warrant further investigation.

## 7 Summary

Taken together, TRPA1 channels play diverse roles in cardiovascular physiology and pathology and are involved in the development of cardiac fibrosis, ischemia-reperfusion injury, and arrhythmogenesis. Unlike previous reviews, this article focuses on the role of TRPA1 in the development of cardiovascular diseases. Over the past few years, research has tended to explore the effects of TRPA1 agonists and antagonists in addition to the role of the Trpa1 gene. The roles of TRPA1 in cardiovascular disorders have been summarized in Table 1.

**Table 1:** The role of TRPA1 in cardiovascular disorders.

Cardiovascular disorders	Roles	Animal models	References
Myocardial infarction/myocardial I/R injury	Protective	Rat, mouse	(34, 35)
	Detrimental	Mouse	(37, 38)
	Neutral	Rat, mouse	(36)
Age-related cardiac fibrosis	Protective	Mouse	(31)
Dilated cardiomyopathy and fibrosis	Protective	Rat	(41)
Right ventricular fibrosis	Protective	Mouse	(42)
Post-MI cardiac fibrosis	Detrimental	Mouse	(43)
Diabatic cardiomyopathy and fibrosis	Detrimental	Rat	(44)
Left ventricular hypertrophy and fibrosis	Detrimental	Mouse	(45)

While targeting TRPA1 channels holds promise as a therapeutic approach for cardiovascular diseases, further research is needed to elucidate their precise roles and regulatory mechanisms. Future studies should focus on validating TRPA1 as a viable therapeutic target and exploring novel strategies to modulate TRPA1 activity for the treatment of cardiovascular diseases. As TRPA1 is expressed in various types of cells and tissues, using conditional Trpa1 gene knockout mouse models may provide more precise insights into its role in cardiovascular pathophysiology. Controversial results were observed by using TRPA1 knockout mouse model and agonists/antagonists. Further studies need to confirm the functional status of TRPA1 channels after administrating their agonists and antagonists as their dosage, frequency, and duration may have distinct effects on the receptor. For instance, TRPA1 can be desensitized when exposed to agonists such as AITC, and as such the observed effects of AITC could be due to desensitization of TRPA1 (63). Furthermore, the effects of TRPA1 agonists/antagonists on cardiac sympathetic and parasympathetic nerve activities warrant further clarification. We should be careful that there is a complicated relationship between TRPA1 and L-type calcium channels. For example, the TRPA1 agonist cinnamaldehyde can block L-type calcium channels (47), and the L-type calcium channel blocker nifedipine can activate TRPA1 (64). Therefore, results from experiments testing TRPA1 agonists and antagonists should be carefully interpreted. Several TRPA1 agonists and antagonists have been testing in recent and ongoing clinical trials in various disorders other than cardiovascular diseases (65, 66). When we transform the studies from bench to bedside, the pharmacological effects of TRPA1 agonists and antagonists from animal studies should be carefully interpreted. The therapeutic response to TRPA1 agonists or antagonists may be dependent on the expression level or profile of the TRPA1 protein, therefore, future studies may need to identify specific variants that potentially regulate the expression of TRPA1 in cardiovascular diseases. Comprehensive clinical trials are warranted to evaluate the safety and efficacy of TRPA1-targeted therapies in patients with cardiovascular disorders, paving the way for the development of innovative treatments for these debilitating conditions.

## References

[1] Jordt SE Bautista DM Chuang HH McKemy DD Zygmunt PM Hogestatt ED Mustard oils and cannabinoids excite sensory nerve fibres through the TRP channel ANKTM1. Nature. (2004) 427(6971):260–5. 10.1038/nature02282 14712238

[2] Wang Z Ye D Ye J Wang M Liu J Jiang H The TRPA1 channel in the cardiovascular system: promising features and challenges. Front Pharmacol. (2019) 10:1253. 10.3389/fphar.2019.01253 31680989 PMC6813932

[3] Gao S Kaudimba KK Guo S Zhang S Liu T Chen P Transient receptor potential ankyrin type-1 channels as a potential target for the treatment of cardiovascular diseases. Front Physiol. (2020) 11:836. 10.3389/fphys.2020.00836 32903613 PMC7438729

[4] Pozsgai G Bodkin JV Graepel R Bevan S Andersson DA Brain Sd. Evidence for the pathophysiological relevance of TRPA1 receptors in the cardiovascular system *in vivo*. Cardiovasc Res. (2010) 87(4):760–8. 10.1093/cvr/cvq118 20442136

[5] Meents JE Ciotu CI Fischer MJM. TRPA1: a molecular view. J Neurophysiol. (2019) 121(2):427–43. 10.1152/jn.00524.2018 30485151

[6] Ma S Yu H Zhao Z Luo Z Chen J Ni Y Activation of the cold-sensing TRPM8 channel triggers UCP1-dependent thermogenesis and prevents obesity. J Mol Cell Biol. (2012) 4(2):88–96. 10.1093/jmcb/mjs001 22241835

[7] Karashima Y Talavera K Everaerts W Janssens A Kwan KY Vennekens R TRPA1 acts as a cold sensor *in* vitro and in vivo. Proc Natl Acad Sci U S A. (2009) 106(4):1273–8. 10.1073/pnas.0808487106 19144922 PMC2633575

[8] Moparthi L Sinica V Moparthi VK Kreir M Vignane T Filipovic MR The human TRPA1 intrinsic cold and heat sensitivity involves separate channel structures beyond the N-ARD domain. Nat Commun. (2022) 13(1):6113. 10.1038/s41467-022-33876-8 36253390 PMC9576766

[9] Pan Y Thapa D Baldissera L Jr Argunhan F Aubdool AA Brain SD. Relevance of TRPA1 and TRPM8 channels as vascular sensors of cold in the cutaneous microvasculature. Pflugers Arch. (2018) 470(5):779–86. 10.1007/s00424-017-2085-9 29164310 PMC5942358

[10] Aubdool AA Graepel R Kodji X Alawi KM Bodkin JV Srivastava S TRPA1 is essential for the vascular response to environmental cold exposure. Nat Commun. (2014) 5:5732. 10.1038/ncomms6732 25501034 PMC4284811

[11] Miyake T Nakamura S Zhao M So K Inoue K Numata T Cold sensitivity of TRPA1 is unveiled by the prolyl hydroxylation blockade-induced sensitization to ROS. Nat Commun. (2016) 7:12840. 10.1038/ncomms12840 27628562 PMC5027619

[12] Bautista DM Jordt SE Nikai T Tsuruda PR Read AJ Poblete J TRPA1 mediates the inflammatory actions of environmental irritants and proalgesic agents. Cell. (2006) 124(6):1269–82. 10.1016/j.cell.2006.02.023 16564016

[13] Talavera K Startek JB Alvarez-Collazo J Boonen B Alpizar YA Sanchez A Mammalian transient receptor potential TRPA1 channels: from structure to disease. Physiol Rev. (2020) 100(2):725–803. 10.1152/physrev.00005.2019 31670612

[14] Xiao R Zhang B Dong Y Gong J Xu T Liu J A genetic program promotes C. elegans longevity at cold temperatures via a thermosensitive TRP channel. Cell. (2013) 152(4):806–17. 10.1016/j.cell.2013.01.020 23415228 PMC3594097

[15] Ma S Wang HD. Knockout of Trpa1 exacerbates renal ischemia-reperfusion injury with classical activation of macrophages. Am J Hypertens. (2021) 34(1):110–6. 10.1093/ajh/hpaa162 33005917

[16] Ma S Zhang Y He K Wang P Wang HD. Knockout of TRPA1 exacerbates angiotensin II-induced kidney injury. Am J Physiol Renal Physiol. (2019) 317(3):F623–31. 10.1152/ajprenal.00069.2019 31339777

[17] Koivisto AP Belvisi MG Gaudet R Szallasi A. Advances in TRP channel drug discovery: from target validation to clinical studies. Nat Rev Drug Discov. (2022) 21(1):41–59. 10.1038/s41573-021-00268-4 34526696 PMC8442523

[18] Andrei SR Sinharoy P Bratz IN Damron DS. TRPA1 is functionally co-expressed with TRPV1 in cardiac muscle: co-localization at z-discs, costameres and intercalated discs. Channels (Austin). (2016) 10(5):395–409. 10.1080/19336950.2016.1185579 27144598 PMC4988441

[19] Oguri G Nakajima T Kikuchi H Obi S Nakamura F Komuro I. Allyl isothiocyanate (AITC) activates nonselective cation currents in human cardiac fibroblasts: possible involvement of TRPA1. Heliyon. (2021) 7(1):e05816. 10.1016/j.heliyon.2020.e05816 33458442 PMC7797518

[20] Paulsen CE Armache JP Gao Y Cheng Y Julius D. Structure of the TRPA1 ion channel suggests regulatory mechanisms. Nature. (2015) 520(7548):511–7. 10.1038/nature14367 25855297 PMC4409540

[21] Suo Y Wang Z Zubcevic L Hsu AL He Q Borgnia MJ Structural insights into electrophile irritant sensing by the human TRPA1 channel. Neuron. (2020) 105(5):882–94.e5. 10.1016/j.neuron.2019.11.023 31866091 PMC7205012

[22] Cordero-Morales JF Gracheva EO Julius D. Cytoplasmic ankyrin repeats of transient receptor potential A1 (TRPA1) dictate sensitivity to thermal and chemical stimuli. Proc Natl Acad Sci U S A. (2011) 108(46):E1184–91. 10.1073/pnas.1114124108 21930928 PMC3219143

[23] Bessac BF Sivula M von Hehn CA Escalera J Cohn L Jordt ES. TRPA1 is a major oxidant sensor in murine airway sensory neurons. J Clin Invest. (2008) 118(5):1899–910. 10.1172/JCI34192 18398506 PMC2289796

[24] Bandell M Story GM Hwang SW Viswanath V Eid SR Petrus MJ Noxious cold ion channel TRPA1 is activated by pungent compounds and bradykinin. Neuron. (2004) 41(6):849–57. 10.1016/s0896-6273(04)00150-3 15046718

[25] Macpherson LJ Dubin AE Evans MJ Marr F Schultz PG Cravatt BF Noxious compounds activate TRPA1 ion channels through covalent modification of cysteines. Nature. (2007) 445(7127):541–5. 10.1038/nature05544 17237762

[26] Zhao J Lin King JV Paulsen CE Cheng Y Julius D. Irritant-evoked activation and calcium modulation of the TRPA1 receptor. Nature. (2020) 585(7823):L141–5. 10.1038/s41586-020-2480-9 32641835 PMC7483980

[27] Fine M Li X. Insights into the irritating mechanisms of TRPA1 revealed by cryo-EM. Neuron. (2021) 109(2):194–6. 10.1016/j.neuron.2020.12.017 33476560

[28] Rivera-Mancilla E Al-Hassany L Marynissen H Bamps D Garrelds IM Cornette J. Functional analysis of TRPA1, TRPM3, and TRPV1 channels in human dermal arteries and their role in vascular modulation. Pharmaceuticals (Basel). (2024) 17(2):156. 10.3390/ph17020156 38399371 PMC10892635

[29] Yang Y Wang D Wan J Ran F Yang L Chen S The role of transient receptor potential ankyrin 1 in age-related endothelial dysfunction. Exp Gerontol. (2021) 154:111517. 10.1016/j.exger.2021.111517 34419618

[30] Balestrini A Joseph V Dourado M Reese RM Shields SD Rouge L A TRPA1 inhibitor suppresses neurogenic inflammation and airway contraction for asthma treatment. J Exp Med. (2021) 218(4):e20201637. 10.1084/jem.20201637 33620419 PMC7918756

[31] Ma S Wang DH. Knockout of Trpa1 accelerates age-related cardiac fibrosis and dysfunction. PLoS One. (2022) 17(9):e0274618. 10.1371/journal.pone.0274618 36103570 PMC9473441

[32] Chiong M Wang ZV Pedrozo Z Cao DJ Troncoso R Ibacache M Cardiomyocyte death: mechanisms and translational implications. Cell Death Dis. (2011) 2(12):e244. 10.1038/cddis.2011.130 22190003 PMC3252742

[33] Wakil SM Ram R Muiya NP Mehta M Andres E Mazhar N A genome-wide association study reveals susceptibility loci for myocardial infarction/coronary artery disease in Saudi Arabs. Atherosclerosis. (2016) 245:62–70. 10.1016/j.atherosclerosis.2015.11.019 26708285

[34] Lu Y Piplani H McAllister SL Hurt CM Gross RE. Transient receptor potential ankyrin 1 activation within the cardiac myocyte limits ischemia-reperfusion injury in rodents. Anesthesiology. (2016) 125(6):1171–80. 10.1097/ALN.0000000000001377 27748654 PMC5110384

[35] Andrei SR Ghosh M Sinharoy P Damron DS. Stimulation of TRPA1 attenuates ischemia-induced cardiomyocyte cell death through an eNOS-mediated mechanism. Channels (Austin). (2019) 13(1):192–206. 10.1080/19336950.2019.1623591 31161862 PMC6557600

[36] Hoebart C Kiss A Pilz PM Szabo PL Podesser BK Fischer MJM. TRPA1 As target in myocardial infarction. Int J Mol Sci. (2023) 24(3):2516. 10.3390/ijms24032516 36768836 PMC9917254

[37] Conklin DJ Guo Y Nystoriak MA Jagatheesan G Obal D Kilfoil PJ TRPA1 channel contributes to myocardial ischemia-reperfusion injury. Am J Physiol Heart Circ Physiol. (2019) 316(4):H889–99. 10.1152/ajpheart.00106.2018 30735434 PMC6483018

[38] Li R Liu R Yan F Zhuang X Shi H Gao X. inhibition of TRPA1 promotes cardiac repair in mice after myocardial infarction. J Cardiovasc Pharmacol. (2020) 75(3):240–9. 10.1097/FJC.0000000000000783 31868827

[39] Lu L Guo J Hua Y Huang K Magaye R Cornell J Cardiac fibrosis in the ageing heart: contributors and mechanisms. Clin Exp Pharmacol Physiol. (2017) 44(Suppl 1):55–63. 10.1111/1440-1681.12753 28316086

[40] Biernacka A Frangogiannis NG. Aging and cardiac fibrosis. Aging Dis. (2011) 2(2):158–73. 21837283 PMC3153299

[41] Wang M Zhao M Zheng Z Pan W Zhang J Yin Z TRPA1 deficiency aggravates dilated cardiomyopathy by promoting S100A8 expression to induce M1 macrophage polarization in rats. FASEB J. (2023) 37(6):e22982. 10.1096/fj.202202079RR 37219522

[42] Li W Zhang Z Li X Cai J Li D Du J CGRP derived from cardiac fibroblasts is an endogenous suppressor of cardiac fibrosis. Cardiovasc Res. (2020) 116(7):1335–48. 10.1093/cvr/cvz234 31504241

[43] Li S Sun X Wu H Yu P Wang X Jiang Z TRPA1 promotes cardiac myofibroblast transdifferentiation after myocardial infarction injury via the calcineurin-NFAT-DYRK1A signaling pathway. Oxid Med Cell Longev. (2019) 2019:6408352. 10.1155/2019/6408352 31217840 PMC6537015

[44] Wang M Zhao M Xu S Zheng Z Zhang J Pan W TRPA1 deficiency attenuates cardiac fibrosis via regulating GRK5/NFAT signaling in diabetic rats. Biochem Pharmacol. (2023) 214:115671. 10.1016/j.bcp.2023.115671 37380112

[45] Wang Z Xu Y Wang M Ye J Liu J Jiang H TRPA1 inhibition ameliorates pressure overload-induced cardiac hypertrophy and fibrosis in mice. EBioMedicine. (2018) 36:54–62. 10.1016/j.ebiom.2018.08.022 30297144 PMC6197736

[46] Hooper JS Stanford KR Alencar PA Alves NG Breslin JW Dean JB Nociceptive pulmonary-cardiac reflexes are altered in the spontaneously hypertensive rat. J Physiol. (2019) 597(13):3255–79. 10.1113/JP278085 31077371 PMC6602842

[47] Alvarez-Collazo J Alonso-Carbajo L Lopez-Medina AI Alpizar YA Tajada S Nilius B Cinnamaldehyde inhibits L-type calcium channels in mouse ventricular cardiomyocytes and vascular smooth muscle cells. Pflugers Arch. (2014) 466(11):2089–99. 10.1007/s00424-014-1472-8 24563220

[48] Aubdool AA Kodji X Abdul-Kader N Heads R Fernandes ES Bevan S TRPA1 activation leads to neurogenic vasodilatation: involvement of reactive oxygen nitrogen species in addition to CGRP and NO. Br J Pharmacol. (2016) 173(15):2419–33. 10.1111/bph.13519 27189253 PMC4945766

[49] Jin L Jagatheesan G Lynch J Guo L Conklin DJ. Crotonaldehyde-induced vascular relaxation and toxicity: role of endothelium and transient receptor potential ankyrin-1 (TRPA1). Toxicol Appl Pharmacol. (2020) 398:115012. 10.1016/j.taap.2020.115012 32320793 PMC7375699

[50] Lynch J Jin L Richardson A Jagatheesan G Lorkiewicz P Xie Z Acute and chronic vascular effects of inhaled crotonaldehyde in mice: role of TRPA1. Toxicol Appl Pharmacol. (2020) 402:115120. 10.1016/j.taap.2020.115120 32634517 PMC7485374

[51] Thompson LC Walsh L Martin BL McGee J Wood C Kovalcik K Ambient particulate matter and acrolein co-exposure increases myocardial dyssynchrony in mice via TRPA1. Toxicol Sci. (2019) 167(2):559–72. 10.1093/toxsci/kfy262 30351402

[52] Conklin DJ Haberzettl P Jagatheesan G Kong M Hoyle WG. Role of TRPA1 in acute cardiopulmonary toxicity of inhaled acrolein. Toxicol Appl Pharmacol. (2017) 324:61–72. 10.1016/j.taap.2016.08.028 27592100 PMC5332481

[53] Conklin DJ. Acute cardiopulmonary toxicity of inhaled aldehydes: role of TRPA1. Ann N Y Acad Sci. (2016) 1374(1):59–67. 10.1111/nyas.13055 27152448 PMC4940269

[54] Kurhanewicz N McIntosh-Kastrinsky R Tong H Ledbetter A Walsh L Farraj A TRPA1 mediates changes in heart rate variability and cardiac mechanical function in mice exposed to acrolein. Toxicol Appl Pharmacol. (2017) 324:51–60. 10.1016/j.taap.2016.10.008 27746315 PMC5391294

[55] Kurhanewicz N Ledbetter A Farraj A Hazari M. TRPA1 mediates the cardiac effects of acrolein through parasympathetic dominance but also sympathetic modulation in mice. Toxicol Appl Pharmacol. (2018) 347:104–14. 10.1016/j.taap.2018.03.027 29627347 PMC6220342

[56] Hazari MS Haykal-Coates N Winsett DW Krantz QT King C Costa DL TRPA1 and sympathetic activation contribute to increased risk of triggered cardiac arrhythmias in hypertensive rats exposed to diesel exhaust. Environ Health Perspect. (2011) 119(7):951–7. 10.1289/ehp.1003200 21377951 PMC3223009

[57] Komatsu T Uchida K Fujita F Zhou Y Tominaga M. Primary alcohols activate human TRPA1 channel in a carbon chain length-dependent manner. Pflugers Arch. (2012) 463(4):549–59. 10.1007/s00424-011-1069-4 22222967

[58] Hoebart C Rojas-Galvan NS Ciotu CI Aykac I Reissig LF Weninger WJ No functional TRPA1 in cardiomyocytes. Acta Physiol (Oxf). (2021) 232(4):e13659. 10.1111/apha.13659 33819369 PMC11478933

[59] Silverman HA Tynan A Hepler TD Chang EH Gunasekaran M Li JH Transient receptor potential ankyrin-1-expressing vagus nerve fibers mediate IL-1beta induced hypothermia and reflex anti-inflammatory responses. Mol Med. (2023) 29(1):4. 10.1186/s10020-022-00590-6 36650454 PMC9847185

[60] Kowalski CW Ragozzino FJ Lindberg JEM Peterson B Lugo JM McLaughlin RJ Cannabidiol activation of vagal afferent neurons requires TRPA1. J Neurophysiol. (2020) 124(5):1388–98. 10.1152/jn.00128.2020 32965166 PMC8356782

[61] Birrell MA Belvisi MG Grace M Sadofsky L Faruqi S Hele DJ TRPA1 agonists evoke coughing in Guinea pig and human volunteers. Am J Respir Crit Care Med. (2009) 180(11):1042–7. 10.1164/rccm.200905-0665OC 19729665 PMC2784411

[62] Bazoukis G Stavrakis S Armoundas AA. Vagus nerve stimulation and inflammation in cardiovascular disease: a state-of-the-art review. J Am Heart Assoc. (2023) 12(19):e030539. 10.1161/JAHA.123.030539 37721168 PMC10727239

[63] Akopian AN Ruparel NB Jeske NA argreaves KM. Transient receptor potential TRPA1 channel desensitization in sensory neurons is agonist dependent and regulated by TRPV1-directed internalization. J Physiol. (2007) 583(Pt 1):175–93. 10.1113/jphysiol.2007.133231 17584831 PMC2277224

[64] Fajardo O Meseguer V Belmonte C Viana F. TRPA1 channels: novel targets of 1,4-dihydropyridines. Channels (Austin). (2008) 2(6):429–38. 10.4161/chan.2.6.7126 18971630

[65] Jain SM Balamurugan R Tandon M Mozaffarian N Gudi G Salhi Y Randomized, double-blind, placebo-controlled trial of ISC 17536, an oral inhibitor of transient receptor potential ankyrin 1, in patients with painful diabetic peripheral neuropathy: impact of preserved small nerve fiber function. Pain. (2022) 163(6):e738–47. 10.1097/j.pain.0000000000002470 34490850 PMC9100440

[66] Tomsen N Alvarez-Berdugo D Rofes L Ortega O Arreola V Nascimento W A randomized clinical trial on the acute therapeutic effect of TRPA1 and TRPM8 agonists in patients with oropharyngeal dysphagia. Neurogastroenterol Motil. (2020) 32(6):e13821. 10.1111/nmo.13821 32064725

